# A Novel Head Capsule Labial Gland Lobe in the Black Field Cricket (Orthoptera: Gryllidae)

**DOI:** 10.1093/jisesa/ieaa068

**Published:** 2020-07-22

**Authors:** Monique Campos Pereira, Paul D Cooper

**Affiliations:** 1 Entomology Laboratory, Department of Morphology, Bioscience Institute, UNESP – São, Paulo State University, Botucatu, SP, Brazil; 2 Ecology & Evolution, Research School of Biology, Bld 46, ANU - The Australian National University, Canberra, ACT, Australia

**Keywords:** retrocerebral complex, corpora cardiaca, cricket, hormones, circulation

## Abstract

We describe a pair of labial gland lobes on either side of the retrocerebral complex in the head of the Australian black field cricket, *Teleogryllus commodus* Walker. As the retrocerebral complex includes the corpora cardiaca and corpora allata, hormones secreted by these glands can be absorbed by these lobes. These lobes of the labial gland are connected to the thoracic lobes via a relatively long duct that enters the main duct draining the thoracic lobes. Measurement of the flow rate of dye from head to thorax in the ducts is rapid, suggesting that these glands may serve as a transport system into the thoracic region. Both serotonin and adipokinetic hormone are shown to be present in the lobes near the retrocerebral complex and the ducts of the thoracic lobes, but whether this connection between the head and thorax acts as a hormone transporter is still unclear.

## Graphical Abstract

**Graphical Abstract:**
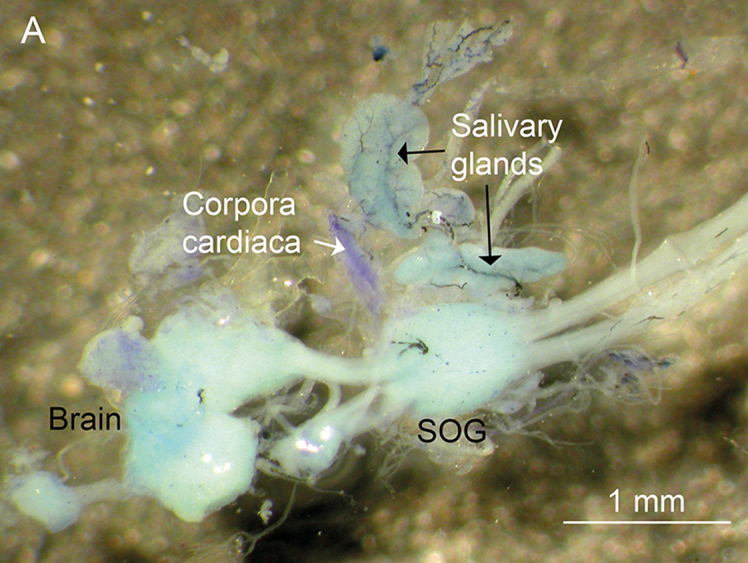
In the black field cricket (*Teleogryllus commodus*), one pair of salivary lobes are located on either side of the corpora cardiaca and corpora allata, connected to the thoracic salivary glands by a long narrow duct. We suggest that this might be one way of transferring hormones from the retrocerebral complex directly into the thoracic region.

Hormone distribution in insects is considered to occur either via the hemolymph from a distant site or locally by neurohemal organs. As the heart of insects is located in the abdomen, with blood being pumped anteriorly towards the head through the dorsal aorta (Douglas and Siva-Jothy 2013), the distribution of hormones through the hemolymph may be limited by its rate of movement, especially in the case of those hormones that are released from the retrocerebral complex (corpora cardiaca and corpora allata). However, many of the processes that require rapid response can be induced by the release from neurohemal organs, or nerves that act to release peptides or amines that act as hormones (Reynolds 2013). The role of both the corpora allata and corpora cardiaca of the retrocerebral complex located in the head are involved in many hormonal activities of insects, so the hemolymph may be the only pathway for transport of their specific hormones.

Glands that are present in the head can be distinguished by location as mandibular, hypopharyngeal, maxillary, and labial (Snodgrass 1935, Simpson 2013). Generally, the labial glands in insects are localized in the thorax and secrete saliva to start the solubilization of food particles. Insects have four different types of labial glands: tubular glands, alveolar (acinar) glands, reservoir glands (Ribeiro 1995), and bilobed glands, the latter restricted to Hemiptera. The labial glands are found in the thorax, although some of the tubular glands extend into the abdomen (Baumann and Walz 2012), and the bilobed glands can be partially located in the head (Ramm et al. 2015).

The alveolar glands have lobes organized like grapes around a branching duct structure (e.g., grasshoppers and cockroaches). Each lobe of the glands is composed of three cell types: the zymogenic cells, parietal (peripheral) cells, and the cells that line the ducts of the glands (Beams and King 1932, Kendall 1969, Lauverjat 1972, Ribeiro 1995). The glands produce saliva for mixing with food by secretion from each lobe that is then conducted to the hypophyrangeal opening by a series of increasingly larger ducts (tertiary at lobes, secondary, and primary before the main salivary duct). Each lobe produces primary saliva by secretion as a result of sodium/potassium secretion in response to stimulation by the amines, serotonin or dopamine, or specific peptides (Smith and House 1979, Vinokurov et al. 2014, Veenstra 2017). The mode of salivary formation appears to be dependent upon the exchange of sodium or potassium with hydrogen via a V-ATPase transporter (Baumann and Walz 2012), with water following from the hemolymph down an osmotic gradient. The ATPase is located on the plasma membrane of the abdominal section of tubular cells (Baumann and Walz 2012) and presumably the parietal cells of the alveolar glands.

While studying the function of thoracic labial glands in the Australian black field cricket, *Teleogryllus commodus* Walker (Orthoptera: Gryllidae), it was noticed that one pair of lobes from these glands came off the main duct just behind the junction of the head and thorax, with the secondary duct curving back around and entering the head region. The lobes connected to the secondary duct were organized on either side of the retrocerebral complex. As most of the labial lobes are present in the thorax, a question arises regarding the need for these ancillary lobes within the head. The presence of labial gland lobes in the head of other insects with alveolar-type glands has not been reported to our knowledge, so the reason for the presence of these lobes in this species is unclear.

The corpora cardiaca store and release hormones, and are connected to the brain by one or two pairs of nerves. This structure releases several different hormones, including adipokinetic hormone (AKH) (Lorenz and Gade 2009). AKH is comprised of 8–10 amino acids and is involved in stimulation of food digestion (Kodrík et al. 2012, Konuma et al. 2012), as well as mobilization of lipids as an energy source (Fukumura et al. 2018). AKH was also reported to stimulate enzymatic activity of polygalacturonase from labial glands of the fire bug (*Pyrrhocoris apterus* L.), suggesting that AKH can be involved in labial gland activity directly (Vinokurov et al. 2014).

In this article, we first describe the location in the head and structure of the labial gland lobes and determine whether these glands are present throughout cricket development. We then asked whether they could act as a transfer system for fluids from the head into the thorax and whether hormones could be translocated from head to thorax, using immunohistochemistry (anti-AKH). As thoracic labial glands are stimulated to secrete saliva in response to serotonin (Ali 1997), we included examination of serotonin in the head lobes to determine whether their secretion may be under similar control to the thoracic lobes.

## Material and Methods

Crickets *T. commodus* were bred in the laboratory under reversed light cycle (12:12 (L:D) h) at 27°C and fed on lettuce, guinea pig lab chow and water ad libitum.

### Dissection of Glands

Adult male crickets (*n* = 10) were removed from the colony injected with methylene blue (0.5% w/v in cricket saline; Cooper and He 1994) 30 min prior to cooling the insect at 4°C. Methylene blue is rapidly absorbed by the glands and therefore enhanced the contrast with the other tissues. Injection of methylene blue followed the methods of Kirby et al. (1984), using a 1 ml syringe and 0.45 mm needle, with the injection through the thorax into the head segment. The head was then viewed using a Wild MZ8 dissecting microscope and dissected to expose the glands and the accompanying nervous tissue and endocrine glands. Images of the brain, subesophageal ganglion, corpora cardiaca, and lobes of labial glands in the head were taken using a Nikon 4500 Coolpix digital camera with an eyepiece adaptor (MM) for the Leica MZ8.

The labial glands within the head were also observed using µ-computer tomography, following fixation with Bouin’s solution and iodine staining (Metscher 2009). The µ computer tomography equipment was developed at the Australian National University, and generates a series of rotational 2-D radiographic images. The relevant parameters are voxel size of 10.9 µm^3^ within a 512 × 512 × 512 matrix, and a voltage of 40 kV at 200 µA. The 2-D images are then imported into Drishti (https://github.com/AjayLimaye/drishti) imaging software for generating the 3-D image.

The retrocerebral complex, including the labial glands, were also dissected out of the head (*n* = 6), placed on a slide under 70% glycerol/30% phosphate-buffered saline (PBS), covered with a coverslip that was sealed in place using nail polish. Images were collected using phase contrast on a Leica DMLB microscope connected to Leica DFC550 digital camera and collected on a Dell Latitude computer. Using the phase contrast image, a diagram of the relationship between the labial glands and retrocerebral complex was drawn from the collected image.

The labial glands stained with methylene blue were dissected free from overlying fat body in the thorax forward along the duct that connected the lobes of the labial glands in the head. Images of the two parts of the labial glands were also photographed with the Nikon 4500 Coolpix camera, but the images had to be stitched together because of the length of the duct connecting the two parts of the labial glands.

Lobes of the thoracic labial gland were isolated along with the adhering fat body and immersed in PBS. The lobes and fat body were placed in a dissecting dish, covered with PBS, and the image captured using a dissecting scope (Leica F205 microscope with DFC 550 camera) using both transmitted and external lighting.

Weight mass of the glands relative to head or body mass was determined by removing the glands from isolated heads of males after weighing the whole cricket and then the isolated head (±0.1 mg) (Mettler AE260). The isolated glands were weighed to the nearest 10^–6^ g (Cahn C30 microbalance).

### The Effect of Age as Indicated by Head Capsule Width on the Presence of the Labial Glands in the Head

Crickets of known age were removed from the colony and separated into four groups by head capsule width as determined using calipers (Mitutoyo): 0.05–0.14 cm; 0.15–0.24 cm; 0.25–0.34 cm; and 0.35–0.45 cm, with the latter group being just smaller than adults (adult male crickets, 0.55 ± 0.1 cm [*n* = 16]). Ten crickets were used in each group and the methylene blue staining technique presented in section ‘Dissection of Glands’ was used to visualize the presence/absence of the labial glands in the head. Crickets were dissected as in section ‘Dissection of Glands’ and scored for presence/absence of labial glands in the head.

### Light Microscopy

To verify that the cephalic lobes had the typical lobe cell structure, heads (*n* = 5) of *T. commodus* were fixed in Bouin’s fixative overnight (>12 h) and then washed with 70% ethanol to remove the excess fixative. The tissues were dehydrated in ethanol and embedded in wax. The wax blocks containing the heads were sectioned at 7 µm (Leica RM 2155). The sections were placed on a slide, dried, and stained with hematoxylin and eosin. Tissues were mounted using Depex, covered with cover slip and allowed to set overnight. Microphotography was done using brightfield on a Leica DMLB connected to a Leica DFC550 digital camera as done with the phase-contrast imaging.

### Head Capsule Labial Gland and Secretion

Hemolymph movement was determined by estimating the approximate hemolymph mass in crickets (0.2 × fresh weight) (Cooper and Vulcano 1997) and the volume of hemolymph collected from the aorta per second as determined using a capillary tube (5 µl). The aorta was pierced through the intersegmental membrane, just anterior to the thorax and the fluid collected. Four individual measurements were made per cricket (*n* = 8) while recording time (s). Time for filling the capillary varied from 3 to 35 s at room temperature (22°C).

To test the hypothesis that the head capsule labial gland of *T. commodus* is involved in fluid movement from head to thorax, an in situ experiment was done. The head and thorax of adult crickets were dissected to permit observation of the head and thoracic labial glands and ducts. The labial gland of the head capsule was walled in with Vaseline (white petroleum jelly) and methylene blue (0.5% in cricket saline, 5 µl delivered [Ovation pipette, VistaLabs]) was placed on the gland and then continually observed using the Leica MZ8 dissecting microscope with time recorded until the dye appeared in the first ducts within the thorax (bifurcation of duct in thorax) (iPhone 5 stopwatch). Microphotography was done with Nikon Shuttlepix P-400Rv Digital Microscope.

### Measuring Lipid in Insect Hemolymph

As a preliminary to section ‘Immunohistochemistry’, the presence of lipid in the hemolymph was determined, in case a daily pattern of fat release into the hemolymph occurred in response to AKH release from the corpora cardiaca. Male crickets (*n* = 6) were cooled (4°C) for 10 min, then an insect pin (0) was inserted into the intersegmental membrane between the head and thorax and 5 µl of hemolymph collected into a capillary tube (Drummond microcaps). The hemolymph was added to 50 µl PBS (pH 7.2) in an Eppendorf tube, mixed using a vortex (IKA Genius 3) for 3 min and then a 2 µl sample added to the sample cards (Merck) for measurement through an infrared spectrometer (Direct Detect Spectrometer – Merck Millipore). The measurements were done prior to feeding and after one, two, and 24 h following feeding. Pure olein oil was used as a standard for quantification (1 µl added to 200 µl PBS, mixed, and 2 µl added to standard card).

### Immunohistochemistry

Using the information from lipid in the hemolymph, crickets that were starved 1 d were used immunohistochemistry for experiments, as AKH may be released from the corpora cardiaca after a short period of starvation (Fukumura et al. 2018), and therefore may be more likely to be absorbed by the cephalic salivary gland. The method for immunohistochemistry is similar to that used in East et al. (1997) and is briefly summarized.

Crickets were dissected to expose the thorax and head under saline, and the cephalic labial gland, corpora cardiaca, and thoracic labial glands were removed. The tissues were fixed in 4% paraformaldehyde at room temperature for 30 min. Paraformaldehyde was removed by washing with PBS and the tissues made permeable by incubating in methanol (5 min in 70% methanol [MeOH] in PBS, 60 min 100% MeOH, 5 min in 70% MeOH in PBS), followed by PBS washes to remove MeOH. The tissues were washed in PBT (PBS with 0.2% BSA and 0.1% Triton X-100), incubated for 30 min in 100 µl PBT+N (4 ml PBT + 200 µl normal goat serum) and then incubated with the primary antibody (either rabbit anti-serotonin ((Immunostar Lot542021) (diluted 1:2000) or rabbit anti-(Tyr^1^)-adipokinetic hormone (*Locusta migratoria*) (Bachem Peninsula Lot A00630) diluted (1:1000)) in PBT+N overnight at 4°C. The primary antibody was removed by washing with PBT, followed by incubation for 30 min in 100 µl of PBT+N. The tissues were then incubated overnight (4°C) in the secondary antibody (1:300 dilution) (Dylight 549 – conjugated AffiniPure goat anti-rabbit IgG (H+L)) (Jackson ImmunoResearch). Tissues were finally washed in PBT, followed by PBS and mounted on slides in 70% glycerol in PBS. Micrographs were taken using an upright fluorescence microscope (Leica DM5500B with a Leica 7000T camera) using optical sectioning (2–3 µm in the *z*-axis) and external focus for generating the final image. Controls for this procedure followed the same steps but omitted the primary antibody to ensure that fluorescence was dependent upon the presence of the primary antibody. Controls were imaged with camera setting at 30% sensitivity in order to show the tissue, as the 12–15% sensitivity used for the antibody-included tissues yielded only black images.

### Statistical Analysis

Statistics were calculated using JMP 13 (SAS) and reported as mean ± 1 standard error, with the number of measurements in parentheses, except in Table 2, where n is given in the legend. Comparison of fed and starved crickets for the movement of methylene blue from the head into the thorax was done using an analysis of variance (ANOVA).

## Results

### Gland Structure

**Fig. 1.**
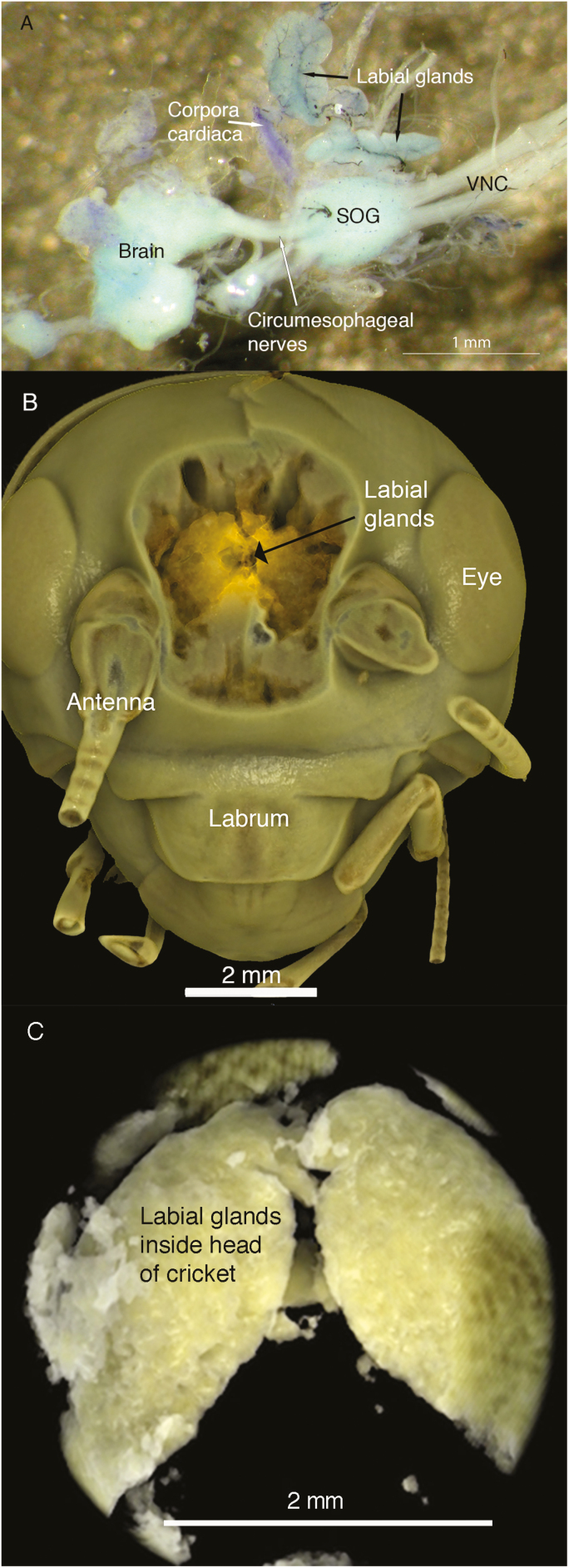
(A) Dissection of labial gland, corpora cardiac, and nervous system from head of cricket following staining with methylene blue. SOG, subœsophageal ganglion. (B) In situ localization of labial glands using microcomputer tomography (µCT) following staining with iodine. (C) Expansion of glands from µCT image indicating the pair of glands present within the head.

**Fig. 2.**
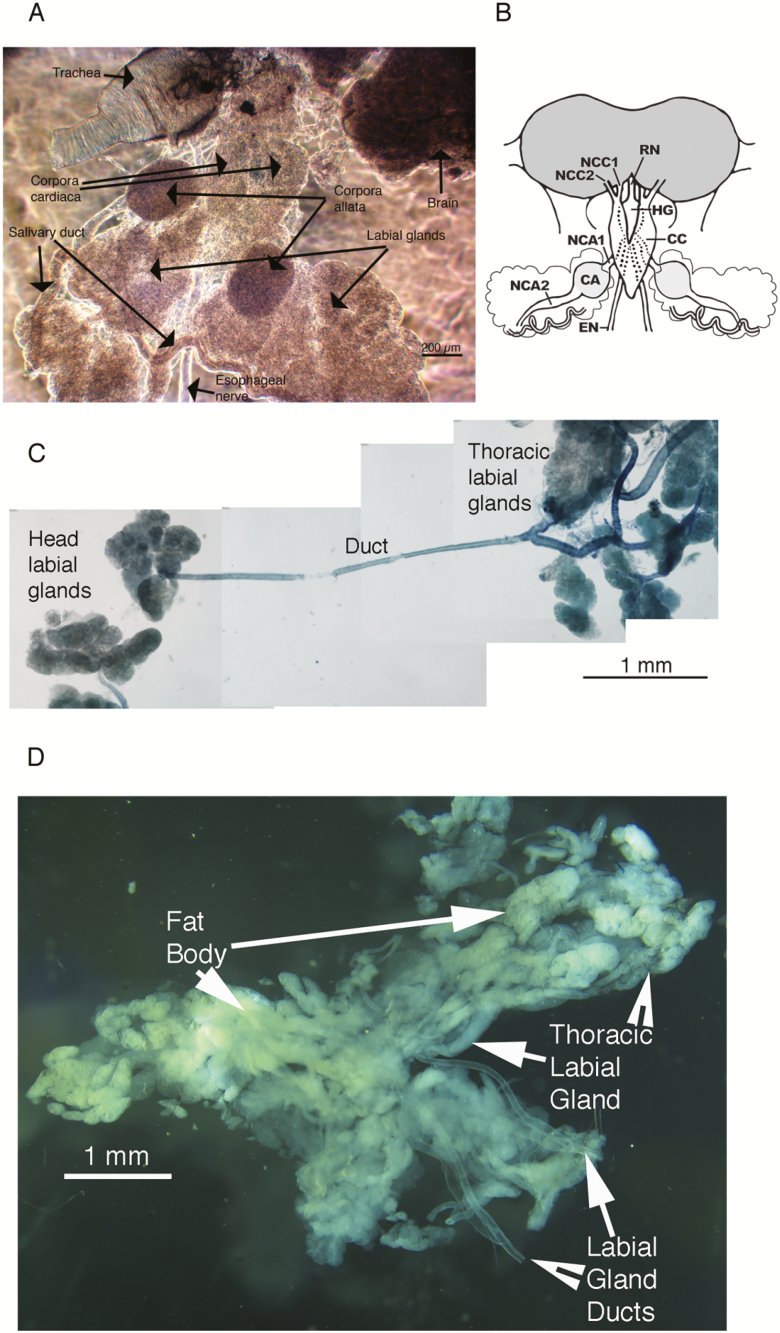
(A) Phase contrast image of the retrocerebral complex with associated labial gland near the corpus allatum. (B) Diagram of the retrocerebral complex (corpora cardiaca (CC), corpora allata (CA) and hypocerebral ganglia (HG)) with the labial gland lobes on either side. NCA1= *nervus corporis allati* 1; NCC1= *nervi corporis cardiaci* 1; NCC2 = *nervi corporis cardiaci* 2; RN = recurrent nerve that runs from frontal ganglion to hypocerebral ganglion (figure redrawn from Pipa and Moore (1988)). (C) Relationship of head labial gland on left with thoracic labial gland on right, indicating length of ductal connection between these glands. (D) Isolated thoracic labial gland lobes and associated fat body. The fat body is present on dorsal surface of labial gland in the thorax.

The presence of a pair of lobes of the labial gland in the head was confirmed by both the dissections and the µ computer tomography (Fig. 1A–C) (see Supp Movie [online only]). The glands lie on either side of the retrocerebral complex (Fig. 2A) and connect to the main duct in the thorax near the junction of the head and thorax. The paired lobes are present lateral to the corpora allata and slightly below the corpora cardiaca (Fig. 2B). The extent of the duct that connects this gland to the thoracic labial glands is shown in Fig. 2C. The thoracic lobes of the labial glands lie in the thorax, typically covered by fat body (Fig. 2D). The mean mass of the labial gland lobes present in the head was 517 ± 77.8 µg (*n* = 13), the head mass was 64 ± 2.4 mg (*n* = 6) and the whole cricket weighed 686 ± 28.2 mg (*n* = 13). The lobes in the head were therefore 0.8 ± 0.16% of head mass (*n* = 6) and 0.07 ± 0.01% of body mass (*n* = 13).

**Fig. 3.**
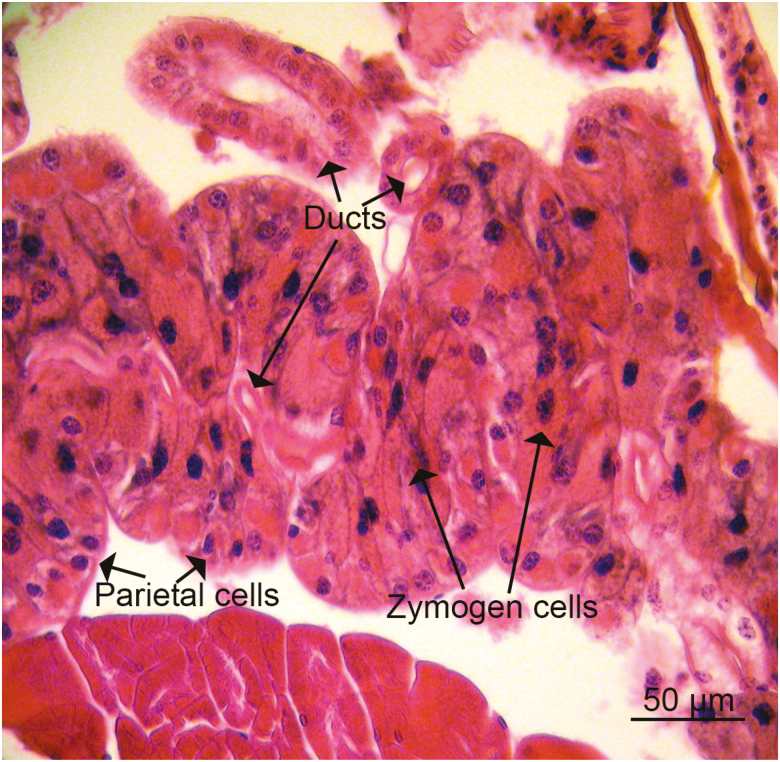
Section of lobe of labial gland from head showing the cellular components (parietal cells, zymogen cells, and ducts and duct cells) and their organization relative to each other.

The structure of the cephalic lobes is typical of the alveolar glands of other insects, as well as the structure of the thoracic glands of crickets (Othman and Cooper, unpublished). Each lobe is composed of zymogenic cells on the inner part of the gland, parietal cells on the outer part of the gland and tertiary ducts interspersed throughout the gland (Fig. 3).

The presence of lobes of labial glands in the head capsule of *T. commodus* was confirmed in all crickets, regardless of age, as estimated from head capsule measurement (Table 1).

**Table 1.** Gland presence with increasing age as indicated by increasing head capsule size of crickets

Head capsule width (mm)	Presence of labial gland
0.5–1.4	10
1.5–2.4	10
2.5–3.4	10
3.5–4.5	10

Age is indicated by growth of head capsule width. *n* = 10 for each age group.

### Hemolymph and Gland Fluid Movements

Male crickets had a mean mass of 647 ± 25.1 mg, which would give an approximate hemolymph volume of 130 ± 5.0 µl hemolymph (calculated as described in section ‘Head Capsule Labial Gland and Secretion’). Mean blood flow through the aorta was 0.7 ± 0.04 µl/s, suggesting that blood could be circulated every 3 min (130 µl/0.7 µl/s) in crickets if circulation was a linear movement. Oleic acid equivalents were high after 3 d of starvation prior to feeding, decreased within 1 h of feeding and began increasing after 24 h post-feeding (Table 2).

**Table 2.** Changes in oleic acid equivalents (mean ± 1 SE) in crickets starved 3 d, then fed

Time (h)	Oleic acid equivalents
0	1 ± 0.24
1	0.3 ± 0.05
2	0.25 ± 0.05
24	0.51 ± 0.06

Hemolymph samples were taken before feeding (Time 0), 1 and 2 h after feeding and then at 24 h post-feeding. *n* = 6. One oleic acid equivalent is equal to 1 µl oleic acid/200µl PBS.

**Fig. 4.**
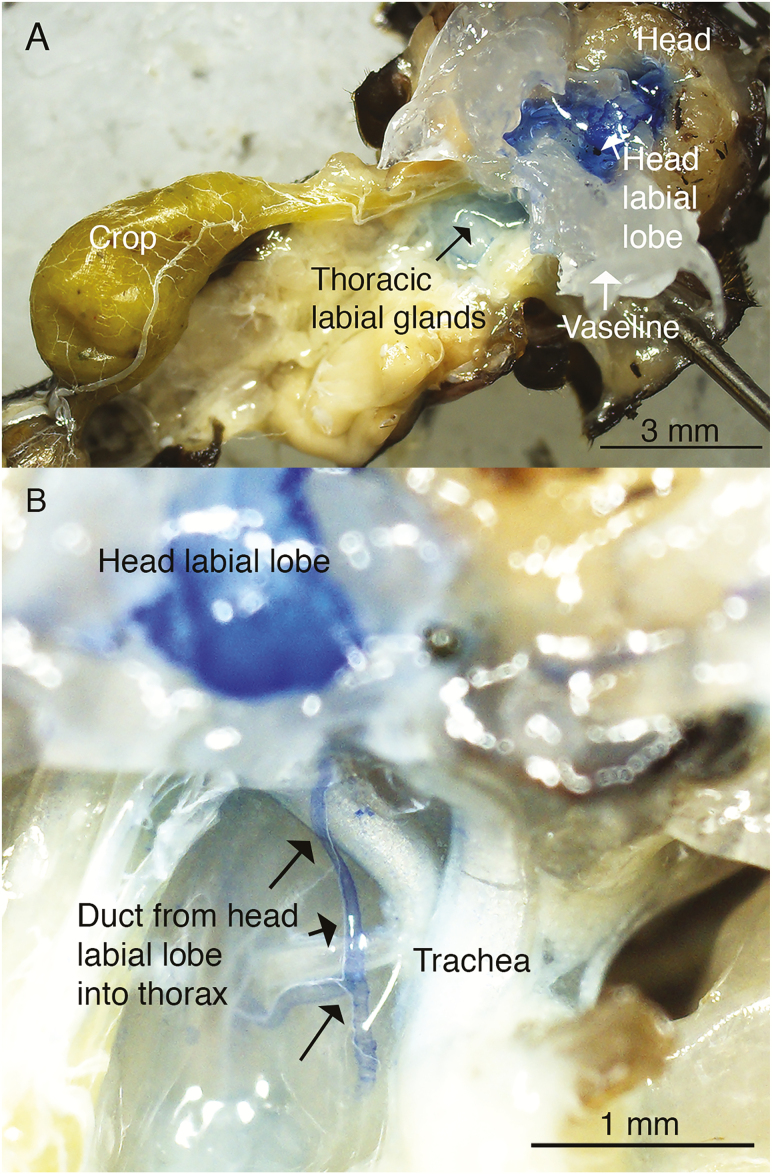
(A) Whole animal preparation showing the isolation of the head labial gland following the introduction of methylene blue solution. (B) Higher magnification of preparation in (A), showing the duct coming from the head labial gland that enters the thoracic labial glands. Notice the bifurcation of the duct (bottom arrow) to supply other lobes of the thoracic labial glands.

To understand how water-soluble hormones could move from head to thoracic lobes of labial glands, methylene blue in saline was used to follow the movement from head to thorax. Soon after the addition of methylene blue to the Vaseline saline reservoir, the stain was absorbed by the cephalic lobe. Fluid containing the methylene blue could be observed moving from the cephalic lobe, along the secondary duct and merging into the thoracic secondary duct (time for movement was 108 ± 11.1 s, *n* = 18) (Fig. 4A and B). However, if the crickets were separated depending upon food in the crop, a significant difference was found in time for fluid movement to the thoracic between crickets with food present in the crop (136 ± 12.7 s) and those without food in the crop (79 ± 12.7 s) (*F*_[1,16]_ = 10.04, *P* = 0.006).

### Gland Immunohistochemisty

**Fig. 5.**
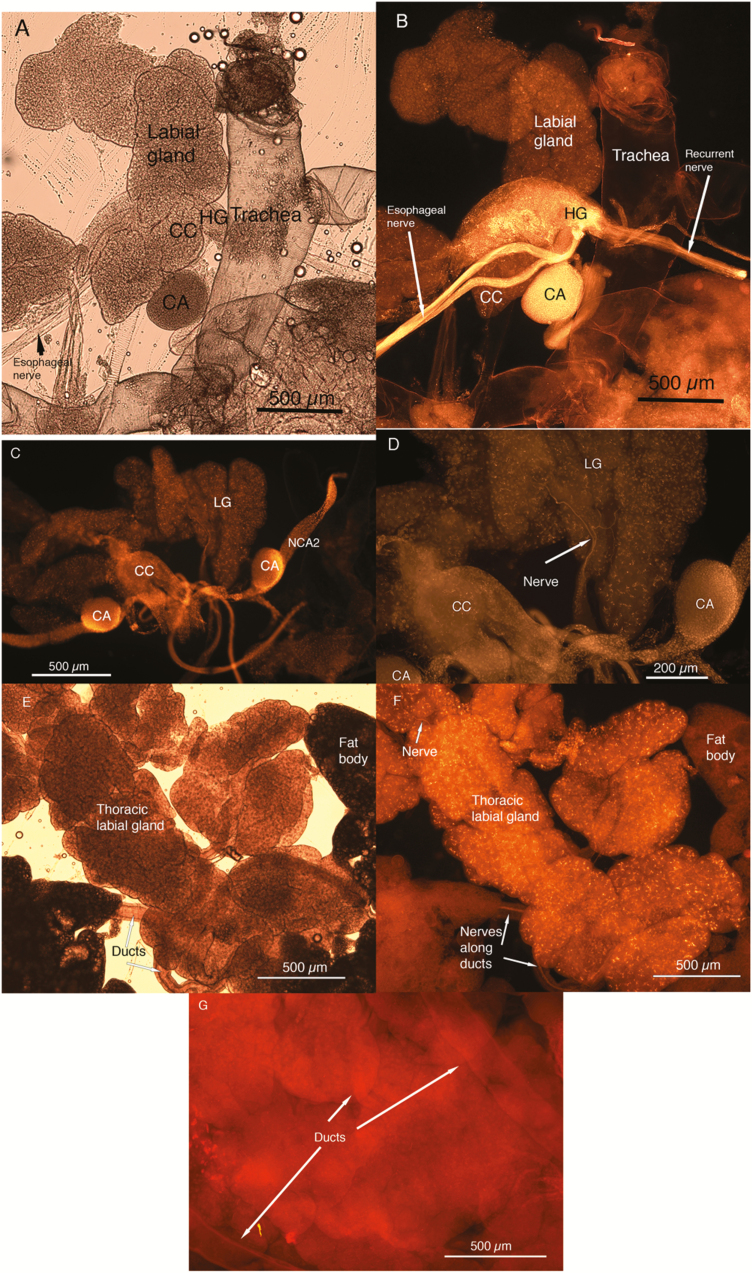
(A and B) Differential interference image (DIC) and fluorescent image of retrocerbral complex (RC) stained with antibody for serotonin and Dylight 597 secondary antibody. The recurrent and esophageal nerves contain serotinergic fibers. (C and D) The CA have many fibers running over the surface, with the labial glands having nerves entering and extending over the surface of the glands. The labial glands also have many cells and some nerves that are serotinergic. (E and F) The thoracic labial glands have a similar pattern of staining as the labial glands in the head, although the ducts have nerves that run along their lengths. (G) nonspecific staining in control (camera sensitivity was increased to 30% from 15% to show the tissue).

The presence of serotonergic cells and nerves in the labial glands within the head was extensive (Fig. 5A and B). The *nervus corporis allati* 2 (NCA2) were clearly stained (Fig. 5C), and there were serotinergic nerves that innervated the labial glands (Fig. 5D). Staining for serotonin did not seem to vary between the zymogenic and parietal cells. Both the corpora allata surfaces and the corpora cardiaca were positive for serotonin, as were nerves within both the recurrent and esophageal nerves.

The labial glands of the thorax showed similar staining patterns as the labial glands of the head, but the ducts clearly had positive staining nerves that ran along the ducts (Fig. 5E and F). The negative control showed no specific staining pattern and required a greater sensitivity to record a visible image (Fig. 5G).

**Fig. 6.**
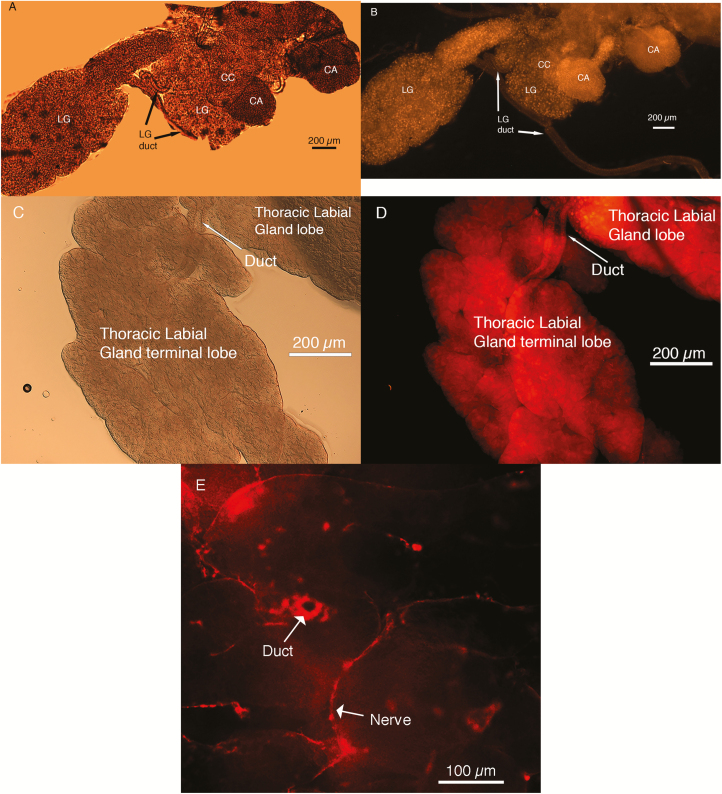
(A and B) DIC and fluorescent images of RC stained for AKH and Dylight 597 secondary antibody. Staining shows labial gland, corpora allata surface, and corpora cardiaca all positive for AKH hormone. (C and D) DIC and fluorescent image of thoracic labial gland stained for AKH and Dylight 597 secondary antibody. Presence of AKH appears to be within the ducts, although staining does appear to be present in some of the glands as well extending towards edge of gland. (E) Single-layer image of thoracic labial gland that shows one duct and several nerves stained.

The CC and CA were both positive for AKH (Fig. 6A and B), and AKH appeared to be dispersed throughout the labial glands. However, AKH in the labial lobe ducts does not appear to be present (Fig. 6B). The thoracic lobes of the labial gland were positive for the presence of the antibody (Fig. 6C and D), with both ducts and nerves positive for the antibody (Fig. 6E).

## Discussion

The presence of lobes of an alveolar labial gland within the head capsule connected to the thoracic gland has not been reported previously for any insect as far as we know. As the gland is present in all ages of crickets, the role of the gland is not dependent upon cricket age or size, but increases in size as the crickets grow and may be part of the retrocerebral complex because of the location. This location of the gland suggests that chemicals could be transferred along the ducts into the pharynx or into the thorax depending upon the fluid pathway. The presence of AKH within the corpora cardiaca, cephalic, and thoracic labial glands suggests that there is a normal transfer occurring from head to thorax, but whether that movement occurs when these insects are feeding, or when normal secretion of the thoracic glands into the pharynx is proceeding is unknown. As time of movement for the dye is reduced in crickets with little food in the crop, and the changes in lipid in the hemolymph relative to time of feeding suggests that transport of a hormone, such as AKH, could be relatively rapid under those conditions.

The movement of dye from head to thorax supports that there is a preferred pathway for fluid movement from head to thorax, but again the animals that we used did not have any large deviation in the fluid movement from head to thorax. The longer time of movement for the dye for crickets with food in the crop suggests that thoracic fluid movement toward the head could delay the transport of material from head to thorax. Whether the pressure exerted by the anterior movement of fluid from thoracic glands can completely block the posterior movement of fluid from the cephalic glands requires further study.

Using the morphology of the gland (radius and length of ducts), we calculated both the volume that the ducts could hold and the ratio of flow for the two regions using Fig. 2B. The duct between the head gland and the thorax held around 1 µl, while the volume of ducts near the thoracic region was about 4.5 times greater. If we considered the flow rates using a pipe that takes into account the ductal surface area such as used in blood flow (Hagen-Poiseuille equation; Hill et al. 2016), and assuming pressure is equal in the two glands, the thoracic gland duct would have 70 times greater fluid movement than the duct from the head. Thus, if the thoracic glands are able to move fluid to the duct from the head, a large reduction in flow from the head would be possible. We did measure a slower flow from animals that had food in the crop and that would be consistent with the dye only being able to diffuse into the thoracic ducts rather than convection flow from the cephalic gland into the thoracic gland. As the thoracic glands appear to be less active when animals are not feeding, this may allow for more fluid to be transferred when animals have less food in the crop.

The control of the cephalic labial gland lobes appears to be through a similar pathway as has been reported in other alveolar glands, as the presence of serotonin was consistent with that observed for cockroaches (Baumann et al. 2002), locusts (Ali 1997), and yellow-winged grasshoppers (Wahida and Cooper 2014). However, we cannot be certain that serotonin does stimulate fluid secretion within the cephalic lobes, as we have not performed that experiment. Potentially, serotonin could have a different effect on the cephalic lobes compared with the thoracic lobes. The origin of the nerves was not clear, as the nerves could have emerged from the hypocerebral ganglion or one of the nerves entering or leaving the ganglion. It may be possible that nerves could be via nerve 7b of the subesophageal ganglion as occurs in thoracic glands (Baines et al. 1989). However, the lack of nerves running along the ducts that extend to the thoracic lobes makes this location of innervation unlikely. Possibly, the termination of NCA2 could release serotonin to activate the lobes nearby, as Pipa and Moore (1988) suggested that that region could act as a neurohemal area as well. An alternative pathway of control would be as suggested above, completely dependent upon the change in secretion and flow from the thoracic lobes.

The presence of AKH-positive nerves in the thoracic glands may indicate that AKH has a role in labial gland function. An increase in salivary enzyme activity is observed when AKH is present in *Pyrrocoris apterus* L. (Vinokurov et al. 2014). However, if feeding could stimulate the movement of AKH or the nerves that are present could be stimulated to initiate enzyme release from the zymogenic cells, then that might be a component of salivary stimulation. The thoracic labial glands become smaller over 3 d of starvation but swell to normal size within 10 min of feeding (Othman and Cooper in preparation), a response that does not appear to be controlled by amines. Further work is needed to clarify the role that peptides and amines have in controlling both the thoracic and cephalic lobes of the labial glands.

Although the control system for the thoracic glands is unclear, the cephalic labial gland could have an endocrine circulating function. The movement of fluid appears to be within minutes from the cephalic to thoracic glands, but how any material would then move out of the thoracic glands is unknown. Calculations for normal circulation also suggest that hormones could be transferred in minutes, but that only would occur if hemolymph moved linearly, such as occurs through the aorta of the insect. As studies measuring hemolymph volume with ^14^C-inulin require 1–2 h for equilibrium (Wharton 1965, Cooper and Vulcano 1997), the time for hormonal distribution is more likely within 0.5–1 h. As many of the responses of insects are much faster than that, alternative modes of hormone distribution are likely, as occurs with neurohemal organs, and the cephalic labial gland and duct system may be another alternative hormonal transport system.

## Supplementary Material

ieaa068_suppl_Supplementary_Movie

## Data Availability

Data from this study are available from the ANU Data Commons: https://datacommons.anu.edu.au/DataCommons/item/anudc:6069 (doi:10.25911/5ee964265b3b6).
